# Clinical consequences of upfront pathology review in the randomised PORTEC-3 trial for high-risk endometrial cancer

**DOI:** 10.1093/annonc/mdx753

**Published:** 2017-11-27

**Authors:** S M de Boer, B G Wortman, T Bosse, M E Powell, N Singh, H Hollema, G Wilson, M N Chowdhury, L Mileshkin, J Pyman, D Katsaros, S Carinelli, A Fyles, C M McLachlin, C Haie-Meder, P Duvillard, R A Nout, K W Verhoeven-Adema, H Putter, C L Creutzberg, V T H B M Smit, for PORTEC Study Group

**Affiliations:** 1 Department of Radiation Oncology, Leiden University Medical Center, Leiden, The Netherlands;; 2 Department of Pathology, Leiden University Medical Center, Leiden, The Netherlands;; 3 Department of Clinical Oncology, Barts Health NHS Trust, St Bartholomew’s Hospital, London;; 4 Department of Cellular Pathology, Barts Health NHS Trust, Royal London Hospital, London, UK;; 5 Department of Pathology, University Medical Center Groningen, Groningen, The Netherlands;; 6 Department of Pathology, Central Manchester Hospitals NHS Foundation Trust, Manchester Royal Infirmary, Manchester, UK;; 7 Division of Cancer Medicine, Peter MacCallum Cancer Centre, Melbourne, Australia; 8 Department of Anatomical Pathology, Royal Women’s Hospital, Parkville, Australia;; 9 Department of Surgical Sciences, Az O-Universitaria Città della Salute di Torino, Torino, Italy; 10 Division of Pathology and Laboratory Medicine, European Institute of Pathology, Milan, Italy;; 11 CCTG, Radiation Medicine Program, Princess Margaret Cancer Centre, Toronto, Canada; 12 Department of Pathology and Laboratory Medicine, Western University, London, Canada;; 13 Department of Radiation Oncology, Institut Gustave Roussy, Villejuif, France;; 14 Department of Pathology, Institut Gustave Roussy, Villejuif, France;; 15 Central Trials Office, Comprehensive Cancer Center The Netherlands, Leiden, The Netherlands; 16 Department of Medical Statistics, Leiden University Medical Center, Leiden, The Netherlands

**Keywords:** endometrial carcinoma, randomised trial, radiation therapy, chemotherapy, pathology review, high risk

## Abstract

**Background:**

In the PORTEC-3 trial, women with high-risk endometrial cancer (HR-EC) were randomised to receive pelvic radiotherapy (RT) with or without concurrent and adjuvant chemotherapy (two cycles of cisplatin 50 mg/m^2^ in weeks 1 and 4 of RT, followed by four cycles of carboplatin AUC5 and paclitaxel 175 mg/m^2^). Pathology review was required before patient enrolment. The aim of this analysis was to evaluate the role of central pathology review before randomisation.

**Patients and methods:**

A total of 1295 cases underwent pathology review to confirm HR-EC in the Netherlands (*n *=* *395) and the UK (*n *=* *900), and for 1226/1295 (95%) matching review and original reports were available. In total, 329 of these patients were enrolled in the PORTEC-3 trial: 145 in the Netherlands and 184 in the UK, comprising 48% of the total PORTEC-3 cohort of 686 participants. Areas of discrepancies were evaluated, and inter-observer agreement between original and review opinion was evaluated by calculating the kappa value (*κ*).

**Results:**

In the 1226 pathology reviews, 6356 selected items were evaluable for both original and review pathology. In 43% of cases at least one pathology item changed after review. For 102 patients (8%), this discrepancy led to ineligibility for the PORTEC-3 trial, most frequently due to differences in the assessment of histological type (34%), endocervical stromal involvement (27%) and histological grade (19%). Lowest inter-observer agreement was found for histological type (*κ* = 0.72), lymph-vascular space invasion (*κ* = 0.72) and histological grade (*κ* = 0.70).

**Conclusion:**

Central pathology review by expert gynaeco-pathologists changed histological type, grade or other items in 43% of women with HR-EC, leading to ineligibility for the PORTEC-3 trial in 8%. Upfront pathology review is essential to ensure enrolment of the target trial-population, and to avoid over- or undertreatment, especially when treatment modalities with substantial toxicity are involved.

This study is registered with ISRCTN (ISRCTN14387080, www.controlled-trials.com) and with ClinicalTrials.gov (NCT00411138).

Key Message

In the PORTEC-3 trial, central pathology review before randomisation by expert gynaeco-pathologists changed histological items in 43% of HR-EC patients. This led to ineligibility for the PORTEC-3 trial in 8% of patients. Upfront pathology review is recommended for future trials as well as in daily practice to ensure enrolment of the target trial-population and to avoid over- or undertreatment.

## Introduction

Adjuvant treatment of women with endometrial cancer (EC) is based on clinicopathological risk factors, such as histological grade, myometrial invasion, age and lymph-vascular space invasion (LVSI) [1–3]. A minority of patients (15%) have high-risk disease features, which include endometrioid endometrial carcinoma (EEC) of FIGO stage I grade 3 with deep invasion or with substantial LVSI; stage II or III EEC; or non-endometrioid histologies (NEEC) stage I–III [1–4]. For these patients higher risks of distant metastases and EC-related death have been reported, and adjuvant chemotherapy may be considered [5–8].

As these high-risk criteria comprise different features of the pathology diagnosis, reproducibility is essential. Studies of pathology review by expert subspecialty pathologists, however, have shown that evaluation of female reproductive tract pathology had the highest rates of discrepancies between original and review pathology assessment including discrepancies with consequences for treatment [9]. Challenges for pre-treatment pathology review are that review is time-consuming and expensive, that timelines are tight and logistical procedures are complicated.

The PORTEC-3 trial is an international randomised phase III trial of adjuvant therapy in high-risk EC (HR-EC). Women with HR-EC were randomly allocated (1 : 1) to pelvic radiotherapy (RT) alone or RT plus concurrent and adjuvant chemotherapy. Primary end points are overall survival and failure-free survival. To select patients with true HR-EC and avoid unnecessary intensive treatment in lower-risk cases, upfront pathology review was carried out by expert gynaeco-pathologists of the participating groups to confirm HR-EC and eligibility for the study.

The current analysis was done to establish the value of upfront pathology review. The aims were to explore the proportion of patients who were ineligible for the PORTEC-3 trial after pathology review, and to evaluate inter-observer variability between original and review pathology assessments.

## Methods

### Study design and participants

PORTEC-3 is a randomised Intergroup trial led by the Dutch Gynaecological Oncology Group, with participating groups MRC-NCRI (UK), ANZGOG (Australia and New Zealand), MaNGO (Italy), Fedegyn (France) and CCTG (Canada). Surgery comprised hysterectomy with salpingo-oophorectomy. Lymphadenectomy was at the discretion of the participating centres. For serous or clear cell cancers, surgical staging including omentectomy; peritoneal biopsies and lymphadenectomy was recommended.

Details on patient selection and treatment have been described in a previous publication [10]. Eligible patients had EEC of FIGO 2009 stage 1A grade 3 with LVSI; IB grade 3; stage II, IIIA, IIIB_*parametrial*_ or IIIC; or NEEC stage IA–III.

Patients were randomised (1 : 1) to RT (48.6 Gy) or RT plus adjuvant chemotherapy (two cycles of cisplatin 50 mg/m^2^ in weeks 1 and 4 of RT, followed by four cycles of carboplatin AUC5 and paclitaxel 175 mg/m^2^ every 3 weeks).

Written informed consent (IC) was obtained from all patients. The protocol was approved by the Dutch Cancer Society and the Ethics committees. Participating groups obtained their own IRB and ethics approvals and were funded by separate grants.

### Procedures

Each participating group had appointed expert gynaeco-pathologists as reviewers for the study. After surgery, the pathology diagnosis was made by the regional pathologist. In case of HR-EC, all histopathology slides and a copy of the pathology report were sent for pathology review as part of patient management, to confirm HR-EC within 1 week, with the aim to ensure that only true HR-EC cases were informed and enrolled in the trial. If IC was given, pathology review for the PORTEC-3 trial was completed with trial-specific items. Upon consent for storage of tumour tissue for translational research a formalin-fixed paraffin-embedded (FFPE)-block was centrally stored. All other blocks and slides were sent back to the referring centre.

The items for original and review pathology included WHO histological type, grade, depth of myometrial invasion, distance to serosa or serosal breach, LVSI, cervical stromal involvement, involvement of the tubes and/or ovaries and lymph node involvement. Histological type was evaluated as endometrioid, serous, clear cell, mixed (endometrioid with serous/clear cell components), mucinous, or other histologies according to WHO-classification [11]. Mixed tumours were classified as serous or clear cell when this component was at least 25%, otherwise as mixed. Mucinous tumours were grouped with EEC for analysis. Histological grading was done according to WHO [11]. NEEC was considered high grade per definition (grade 3). The differences in histological grading between original and review pathology were evaluated for EEC. Immunohistochemistry (IHC) was carried out only incidentally, at the discretion of the review pathologist and only if FFPE-blocks were available at time of the central review process.

For the current analysis, anonymised original and review pathology reports from both randomised and non-randomised patients in the Netherlands (NL) and the UK (UK) were assessed. These two countries were chosen as they had the largest number of patients in the trial (together 48%) and all pathology reviews had been done at two centres in each country. For the UK patients, the review pathologist provided a short confirmation of HR-EC and eligibility. For the randomised patients, the review report was completed after IC was given.

### Outcomes

Discrepancies between original and central pathology review were assessed as discrepancies with and without change of eligibility for the PORTEC-3 trial. Reasons for non-eligibility were checked by two expert gynaeco-pathologists (TB and NS).

### Statistical analysis

The data were collected in a SPSS database (version 23.0). For the comparison of the pathology items, Cohen’s kappa value (*κ*) was used [12]. For the interpretation of the *κ* values the scale proposed by Landis and Koch was used [13].

Differences between eligible women who were included or declined the study were analysed by the *χ*^2^ test. Items with *P*-values <0.05 were considered significant.

## Results

### Population and compliance

**Figure 1.**
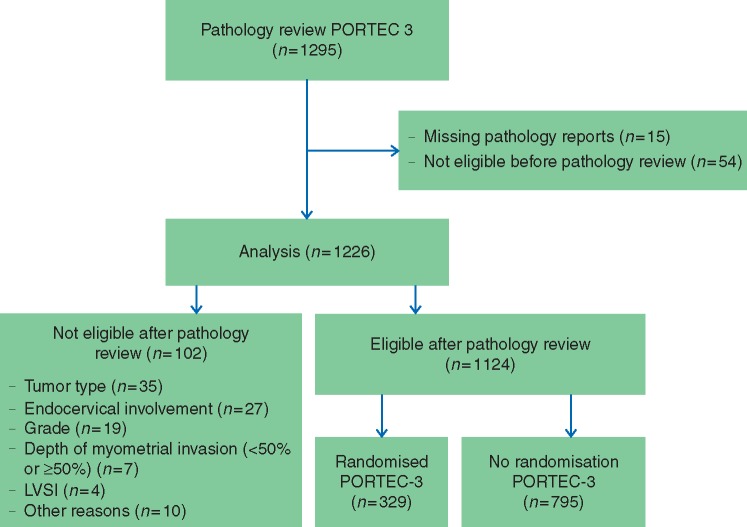
CONSORT diagram.

The PORTEC-3 trial included 686 patients (2006–2013), of whom 145 were recruited in NL and 184 in the UK. Slides from 1295 patients (395 NL, 900 UK) were sent for pathology review. Fifteen original pathology reports (9 NL, 6 UK) were not available for analysis. Fifty-four patients (18 NL, 36 UK) were ineligible based on the original pathology report, which was confirmed by pathology review and they were therefore excluded from the analysis. A total of 1226 patients (368 NL, 858 UK) were eligible based on local pathology and were included in this analysis (see Figure 1, Table 1 and supplementary Table S1, available at *Annals of Oncology* online).

**Table 1.** Major pathology criteria of the eligible patients (*n* = 1226)

Major pathologic criteria		NL patients (*n* = 368)		UK patients (*n* = 858)	
		***n***	**%**	***n***	**%**
Age	<60	100	37%	239	28%
	60–69	110	41%	373	44%
	≥70	58	22%	243	28%
	Missing	100		3	
FIGO stage (2009)	IA	72	20%	138	16%
	IB	93	26%	178	21%
	II	99	27%	263	31%
	IIIA	43	12%	97	12%
	IIIB	18	5%	62	7%
	IIIC	40	11%	101	12%
	Missing	3		19	
Histological type	Endometrioid or mucinous	262	71%	501	59%
	Serous or mixed serous	66	18%	193	23%
	Clear cell or mixed clear cell	31	8%	111	13%
	Other^a^	9	2%	45	5%
	Missing	0		8	
Histological grade	1	81	22%	155	18%
	2	53	14%	135	16%
	3	127	35%	201	24%
	NEEC	107	29%	354	42%
	Missing	0		13	
Myometrial invasion	<50%	135	37%	215	38%
	≥50%	233	63%	346	62%
	Missing	0		297	
Growth through serosa	Yes	21	6%	31	4%
	No	346	94%	675	96%
	Missing	1		152	
Cervical glandular involvement	Yes	135	38%	172	43%
	No	224	62%	230	57%
	Missing	9		456	
Cervical stromal involvement	Yes	138	38%	339	47%
	No	225	62%	382	53%
	Missing	5		137	
LVSI	Yes	198	54%	287	60%
	No	169	46%	194	40%
	Missing	1		377	
Involvement of the ovaries	Yes	46	13%	67	9%
	No	322	87%	666	91%
	Missing	0		125	
Lymph node involvement	Not applicable	252	69%	553	66%
	No malignancy	73	20%	184	22%
	Metastasis	41	11%	101	12%
	Missing	2		20	
Parametrial involvement	Yes	24	13%	61	16%
	No	167	87%	326	84%
	Missing	177		471	

Missing values were not taken into account to the percentages.

The pathology criteria of the NL versus the UK patients were based on review pathology.

a Other histology includes undifferentiated, carcinosarcoma or mixed combinations other than serous/clear cell with endometrioid.

FIGO, International Federation of Gynecology and Obstetrics; LVSI, lymph-vascular space invasion; EEC, endometrioid endometrial cancer; NEEC, non-endometrioid endometrial cancer.

### Discrepancies and inter-observer variability

A total of 6356 pathology items were evaluable for both original and review pathology. For 679 items (11%) there was a discrepancy between original and review pathology. The highest agreement was found for serosal breach (98%) and cervical stromal involvement (94%), and most disagreement for histological type (15%) and grade (20%; see Table 2).

**Table 2.** Inter-observer variability between original and review pathology report for the total cohort

Total cohort									
Pathology item	Total number available for analysis^a^	Missing items	Total discrepancies	Disagreement %^b^	Leading to ineligibility	Leading to ineligibility %^c^	Not leading to ineligibility	Not leading to ineligibility %^d^	*κ* value
Histological type	1217	9	185	15%	35	19%	150	81%	0.72
Histological grade (EEC only)	701	0	139	20%	19	14%	120	86%	0.70
Myometrial invasion	923	304	88	10%	7	8%	81	92%	0.79
Cervical glandular involvement	626	600	73	12%	0	0%	73	100%	0.73
Cervical stromal involvement	1063	163	69	6%	27	39%	42	61%	0.87
LVSI	762	464	101	13%	4	4%	97	96%	0.72
Growth through serosa	1064	162	24	2%	0	0%	24	100%	0.76
Total	6356	1702	679	11%	92	14%	587	86%	NA

a Total number of pathology items available for comparison between original and review pathology.

b Total discrepancies/total number of pathology items available for analysis.

c Number of pathology items leading to ineligibility/total discrepancies.

d Number of pathology items not leading to ineligibility/total discrepancies.

LVSI, lymph vascular space invasion; EEC, endometrioid endometrial cancer.

In 532 cases (43%) at least one pathology item changed after review, which led to ineligibility for the PORTEC-3 trial in 8% (*n *=* *102; Table 3). Most frequent reasons were change of histological type (34%, *n *=* *35), cervical stromal involvement (27%, *n *=* *27) and change of histological grade in 19% (*n *=* *19), which was similar between the NL and UK cohorts. Eighty-three of these 102 became low risk after central pathology review, while in 19 cases the histological type was reclassified as carcinosarcoma; these were therefore still high risk but were not eligible for the PORTEC-3 trial.

**Table 3.** Reasons for ineligibility of 102 patients based on pathological review report

Pathology variables	Cohort (*n* = 102)		NL cohort (*n* = 42)		UK cohort (*n* = 60)	
	*n*	%	*n*	%	*n*	%
Histological type	35	34	14	33	21	35
Histologic grade^a^	19	19	7	17	12	20
Myometrial invasion	7	7	3	7	4	7
Cervical involvement	27	27	12	29	15	25
LVSI	4	4	2	5	2	3
Other^b^	10	10	4	10	6	10
Total ineligible patients	102	100	42	100	60	100
Percentage of total cohort	102	8	42	11	60	7

a Grade shift for endometrioid endometrial carcinoma.

b Other reasons included the absence of involvement of the ovaries, tube or parametrium, or other primary tumour site (cervix, tube or adnex).

LVSI, lymph vascular space invasion; NL, Netherlands; UK, United Kingdom.

Highest rates of inter-observer variability were found for histological type (*κ* = 0.72), LVSI (*κ* = 0.72) and histological grade (*κ* = 0.70; Table 2). See supplementary Table S2, available at *Annals of Oncology* online for results by country and supplementary Figure S1, available at *Annals of Oncology* online. Lowest inter-observer variability was found for cervical stromal invasion (*κ* = 0.87), with overall agreement of 94%. However, a discrepancy here led to ineligibility for the trial in 27/69 (39%) of cases.

Serosal breach was present in only 5% of cases. Although agreement was high for both countries (97% and 99%), *κ* values differed (NL *κ* = 0.83 versus UK *κ* = 0.63), showing that *κ* values are less reliable for items with few observations.

### Histological type and grade

**Figure 2.**
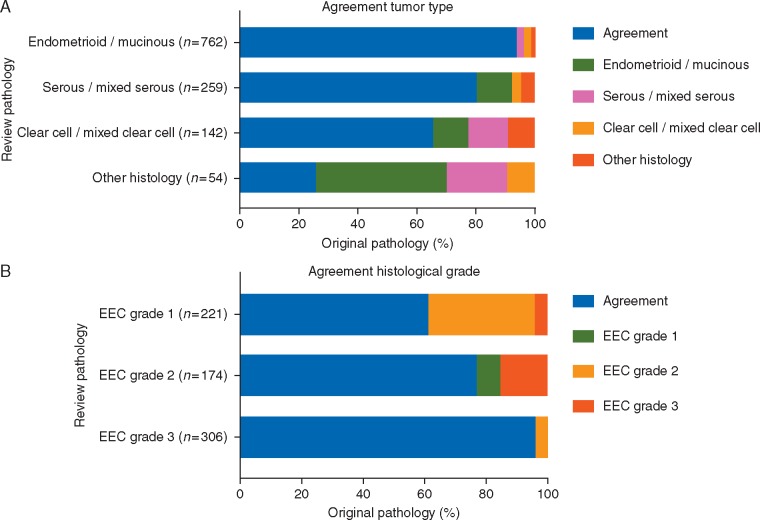
Histological type (A) and histological grade evaluation (B) in original and review pathology.

Figure 2 shows the agreement of histological classification and grade. Overall agreement of histological type was 85%; discrepancies led to ineligibility in 19% of cases (Table 2). Discrepancies were found for all histologies, although the agreement was highest for EEC.

The overall agreement for histological grade was 80%; 16% (*n *=* *113) were downgraded at review pathology, with most frequent shifts (76 cases) from grade 2 to 1. In 4% (*n *=* *26), the grade was higher at review.

## Discussion

In the PORTEC-3 trial of adjuvant RT with or without chemotherapy for women with HR-EC, upfront pathology review was carried out before patient counselling to ensure that only true HR-EC patients were informed about the trial, and that the trial only enrolled true HR-EC cases. The expert gynaeco-pathology review changed the eligibility for 102 women (8%), most frequently due to changes in histological type or cervical stromal involvement. These lower-risk patients did therefore not risk receiving more intensive and potentially toxic treatment. Furthermore, a true HR-EC study population in the PORTEC-3 trial was ensured. For 19 patients the histological type changed to carcinosarcoma and although they were high risk, they were not eligible for the trial.

The inter-observer agreement between original and review pathology was highest for cervical stromal invasion. The most frequent discrepancies were found for histological type, histological grade and presence of LVSI. While many of these discrepancies did not affect eligibility for the current study, they were important for prognosis and adjuvant treatment of patients in clinical practice.

Discrepancies in gynaeco-pathology diagnosis between original and review pathology have been reported before. A Canadian study reported EC as the tumour site with most frequent differences in pathological assessment [14]. Another Canadian cohort reported major discrepancies in 8% of biopsies and hysterectomy specimens taken together, and in 12% of hysterectomy specimens. The most frequent diagnostic discrepancies were assessment of myometrial invasion and histological subtype [15].

In the PORTEC-1 and -2 trials pathology review showed that 24% and 14%, respectively, of patients were in retrospect ineligible, while this was 8% for the PORTEC-3 trial [1, 16, 17]. Eligibility in the PORTEC-1 and -2 studies was determined by grade, myometrial invasion and histological type. Differences in eligibility were often caused by shift of grade 2 to grade 1, while such grade shift did not affect the PORTEC-3 trial where patients had to have either grade 3 or NEEC or advanced stages. Minor discrepancies in grade or histology changed the eligibility for the PORTEC-3 trial in only a minority of patients. However, some shift of grade 2 to grade 1 was seen in the PORTEC-3 trial as well. Previous studies have shown that the intermediate grade is the least reproducible and that a two-tiered grading system assessing high versus low grade would be preferable [18–20]. The lower inter-observer variation in the current study could also reflect the increasing standardisation of pathology criteria and subspecialty training.

Frequent causes of discrepancies were assessment of histological type and cervical involvement. Several studies have addressed challenges in diagnosing serous, clear cell and mixed cancers, the level of agreement varying from 62% to 83% [21–23]. In the study by Han et al. [21], there was consensus on histological type in 72% of cases. With a panel of three IHC markers the agreement increased to 96% [21]. The use of IHC was not routine practice in the period of the PORTEC-3 trial and was only carried out in incidental cases.

Variations in defining cervical stromal involvement have also been reported in a study of 76 cases reviewed by 6 expert gynaeco-pathologists with agreement in only 50%. Difficulties comprised the definition of the junction between the lower uterine segment and the endocervix, and the distinction between unattached tumour components or true cervical stromal involvement [24].

A limitation of this study could be that the pathology reviews took place at four university centres, and inter-observer variations between these gynaeco-pathologists were not assessed. The percentages of major discrepancies were, however, quite similar between the two countries. In the PORTEC-2 trial, higher risk of distant metastasis and lower survival were found for patients who were considered ‘high-risk’ after central review pathology, suggesting that the review pathology was more reliable to predict prognosis when compared with the original pathology [16].

Creating a well-defined trial population with confirmed eligibility by upfront pathology review should be considered the standard for future scientific studies. Expert consultation is being increasingly used, but pathology review might not be part of the standard procedure, because it is time consuming and expensive. To this purpose, further standardisation of pathology criteria, expert education and subspecialisation in gynaeco-pathology are essential, as well as rapid access to expert consultation. The transition to digital pathology will greatly facilitate rapid consultation. Introduction of IHC and molecular analysis using the TCGA molecular subgroup classification will further improve risk assignment [25, 26].

A substantial proportion of eligible women declined participation in the trial, mostly because they did not want to receive chemotherapy. Younger patients and those with a more advanced stage of disease more often consented to participate in the trial (supplementary Table S1, available at *Annals of Oncology* online). The potential treatment consequences for patients should be the main reason to incorporate pathology review in daily practice. In the current study, most patients with discrepancies were downgraded and were spared unnecessary treatment.

In conclusion, upfront pathology review by expert gynaeco-pathologists identified changes in histological type, grade or other items in 43% of patients. Of these, 8% of patients were found ineligible for the trial. This resulted in a true HR-EC population and reliable pathology assessment in the PORTEC-3 trial, which ensures the quality of future translational research. Upfront pathology review is to be preferred in future gynaecological oncology trials and in daily practice. The transition to digital pathology will strongly facilitate rapid expert pathology consultation.

## Supplementary Material

Supplementary Figure S1

Supplementary Table S1

Supplementary Table S2

Supplementary Data

## References

[1] Creutzberg CL, van Putten WL, Koper PC Surgery and postoperative radiotherapy versus surgery alone for patients with stage-1 endometrial carcinoma: multicentre randomised trial. PORTEC Study Group. Post Operative Radiation Therapy in Endometrial Carcinoma. Lancet 2000; 355(9213): 1404–1411. 10791524 10.1016/s0140-6736(00)02139-5

[2] Keys HM, Roberts JA, Brunetto VL A phase III trial of surgery with or without adjunctive external pelvic radiation therapy in intermediate risk endometrial adenocarcinoma: a Gynecologic Oncology Group study. Gynecol Oncol 2004; 92(3): 744–751. 14984936 10.1016/j.ygyno.2003.11.048

[3] Blake P, Swart AM, Orton J Adjuvant external beam radiotherapy in the treatment of endometrial cancer (MRC ASTEC and NCIC CTG EN.5 randomised trials): pooled trial results, systematic review, and meta-analysis. Lancet 2009; 373(9658): 137–146. 19070891 10.1016/S0140-6736(08)61767-5 PMC2646125

[4] Colombo N, Creutzberg C, Amant F ESMO-ESGO-ESTRO consensus conference on endometrial cancer: diagnosis, treatment and follow-up. Radiother Oncol 2015; 117(3): 559–581. 26683800 10.1016/j.radonc.2015.11.013

[5] Straughn JM, Huh WK, Orr JW Jr Stage IC adenocarcinoma of the endometrium: survival comparisons of surgically staged patients with and without adjuvant radiation therapy. Gynecol Oncol 2003; 89(2): 295–300. 12713994 10.1016/s0090-8258(03)00087-8

[6] Greven KM, Randall M, Fanning J Patterns of failure in patients with stage I, grade 3 carcinoma of the endometrium. Int J Radiat Oncol Biol Phys 1990; 19(3): 529–534. 2211200 10.1016/0360-3016(90)90477-2

[7] Creutzberg CL, van Putten WL, Warlam-Rodenhuis CC Outcome of high-risk stage IC, grade 3, compared with stage I endometrial carcinoma patients: the Postoperative Radiation Therapy in Endometrial Carcinoma Trial. JCO 2004; 22: 1234–1241. 10.1200/JCO.2004.08.159 15051771

[8] Bosse T, Peters EE, Creutzberg CL Substantial lymph-vascular space invasion (LVSI) is a significant risk factor for recurrence in endometrial cancer—a pooled analysis of PORTEC 1 and 2 trials. Eur J Cancer 2015; 51(13): 1742–1750. 26049688 10.1016/j.ejca.2015.05.015

[9] Manion E, Cohen MB, Weydert J. Mandatory second opinion in surgical pathology referral material: clinical consequences of major disagreements. Am J Surg Pathol 2008; 32(5): 732–737. 18360282 10.1097/PAS.0b013e31815a04f5

[10] de Boer SM, Powell ME, Mileshkin L Toxicity and quality of life after adjuvant chemoradiotherapy versus radiotherapy alone for women with high-risk endometrial cancer (PORTEC-3): an open-label, multicentre, randomised, phase 3 trial. Lancet Oncol 2016; 17(8): 1114–1126. 27397040 10.1016/S1470-2045(16)30120-6

[11] Tavassoli FA, Devilee P, World Health Organization Classification of Tumours. Pathology and Genetics of Tumours of the Breast and Female Genital Organs. Lyon: IARC Press, 2003.

[12] Cohen J. A coefficient of agreement for nominal scales. Educ Psychol Meas 1960; 20(1): 37–46.

[13] Landis JR, Koch GG. The measurement of observer agreement for categorical data. Biometrics 1977; 33(1): 159–174. 843571

[14] Chafe S, Honore L, Pearcey R, Capstick V. An analysis of the impact of pathology review in gynecologic cancer. Int J Radiat Oncol Biol Phys 2000; 48(5): 1433–1438. 11121644 10.1016/s0360-3016(00)00791-4

[15] Khalifa MA, Dodge J, Covens A Slide review in gynecologic oncology ensures completeness of reporting and diagnostic accuracy. Gynecol Oncol 2003; 90(2): 425–430. 12893212 10.1016/s0090-8258(03)00323-8

[16] Nout RA, Smit VT, Putter H Vaginal brachytherapy versus pelvic external beam radiotherapy for patients with endometrial cancer of high-intermediate risk (PORTEC-2): an open-label, non-inferiority, randomised trial. Lancet 2010; 375(9717): 816–823. 20206777 10.1016/S0140-6736(09)62163-2

[17] Scholten AN, van Putten WL, Beerman H Postoperative radiotherapy for Stage 1 endometrial carcinoma: long-term outcome of the randomized PORTEC trial with central pathology review. Int J Radiat Oncol Biol Phys 2005; 63(3): 834–838. 15927414 10.1016/j.ijrobp.2005.03.007

[18] Scholten AN, Smit VT, Beerman H Prognostic significance and interobserver variability of histologic grading systems for endometrial carcinoma. Cancer 2004; 100(4): 764–772. 14770433 10.1002/cncr.20040

[19] Lax SF, Kurman RJ, Pizer ES A binary architectural grading system for uterine endometrial endometrioid carcinoma has superior reproducibility compared with FIGO grading and identifies subsets of advance-stage tumors with favorable and unfavorable prognosis. Am J Surg Pathol 2000; 24(9): 1201–1208. 10976693 10.1097/00000478-200009000-00002

[20] Alkushi A, Abdul-Rahman ZH, Lim P Description of a novel system for grading of endometrial carcinoma and comparison with existing grading systems. Am J Surg Pathol 2005; 29(3): 295–304. 15725797 10.1097/01.pas.0000152129.81363.d2

[21] Han G, Sidhu D, Duggan MA Reproducibility of histological cell type in high-grade endometrial carcinoma. Mod Pathol 2013; 26(12): 1594–1604. 23807777 10.1038/modpathol.2013.102

[22] Gilks CB, Oliva E, Soslow RA. Poor interobserver reproducibility in the diagnosis of high-grade endometrial carcinoma. Am J Surg Pathol 2013; 37(6): 874–881. 23629444 10.1097/PAS.0b013e31827f576a

[23] Thomas S, Hussein Y, Bandyopadhyay S Interobserver variability in the diagnosis of uterine high-grade endometrioid carcinoma. Arch Pathol Lab Med 2016; 140(8): 836–843. 27139150 10.5858/arpa.2015-0220-OA PMC5656271

[24] McCluggage WG, Hirschowitz L, Wilson GE Significant variation in the assessment of cervical involvement in endometrial carcinoma: an interobserver variation study. Am J Surg Pathol 2011; 35(2): 289–294. 21263250 10.1097/PAS.0b013e3182073ac0

[25] Stelloo E, Nout RA, Osse EM Improved risk assessment by integrating molecular and clinicopathological factors in early-stage endometrial cancer-combined analysis of the PORTEC cohorts. Clin Cancer Res 2016; 22(16): 4215–4224. 27006490 10.1158/1078-0432.CCR-15-2878

[26] Talhouk A, McConechy MK, Leung S Confirmation of ProMisE: a simple, genomics-based clinical classifier for endometrial cancer. Cancer 2017; 123(5): 802–813. 28061006 10.1002/cncr.30496

