# Supplementary figures and images for: Clinical consequences of upfront pathology review in the randomised PORTEC-3 trial for high-risk endometrial cancer
Source: Ann Oncol. 2017 Nov 27;29(2):424–30. doi: 10.1093/annonc/mdx753 (PMC5834053; doi:10.1093/annonc/mdx753)

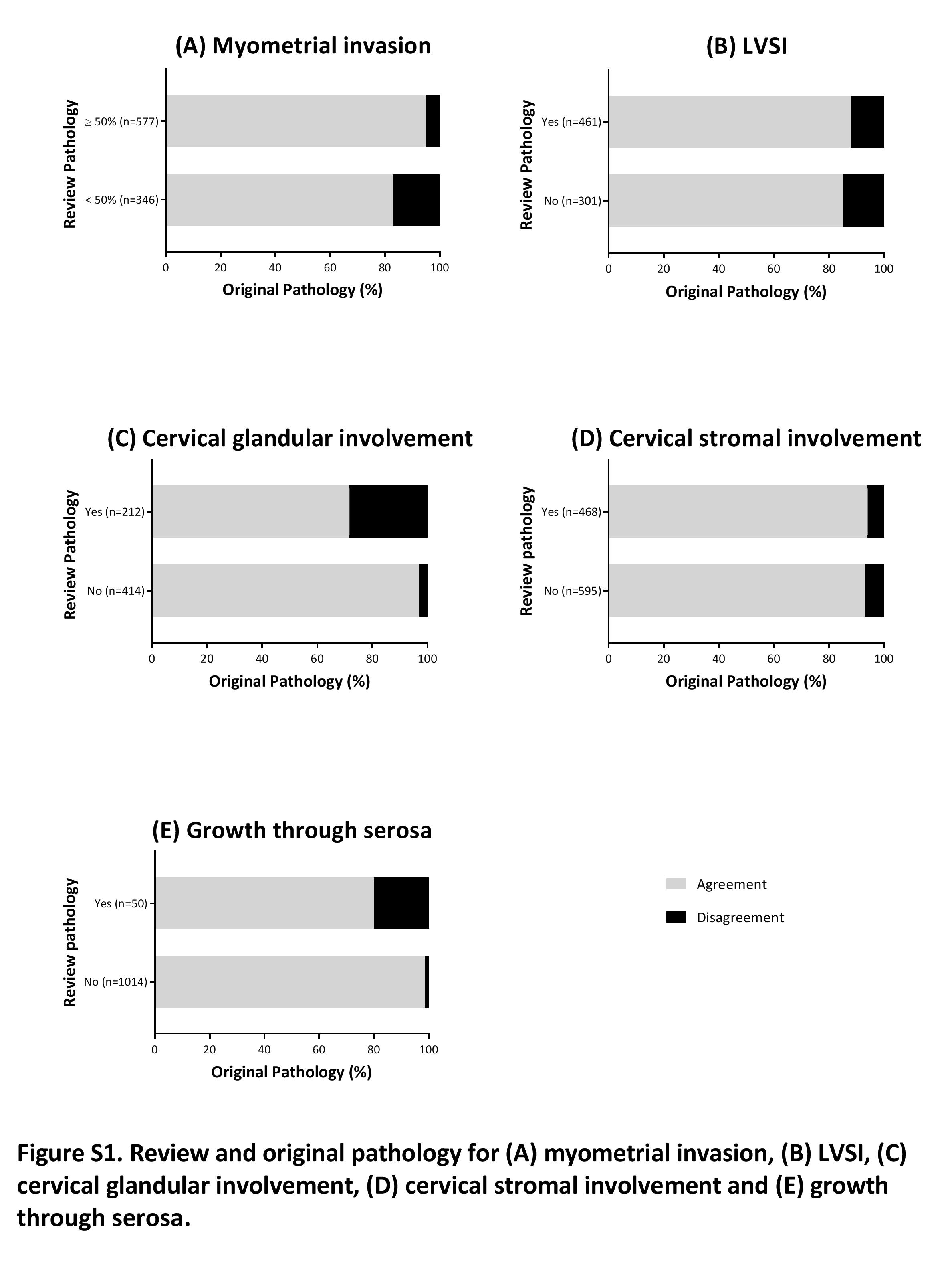

Supplement: Supplementary Figure S1 [file figure_s1_mdx753.jpeg]
