# Supplementary material for: Clinical consequences of upfront pathology review in the randomised PORTEC-3 trial for high-risk endometrial cancer
Source: Ann Oncol. 2017 Nov 27;29(2):424–30. doi: 10.1093/annonc/mdx753 (PMC5834053; doi:10.1093/annonc/mdx753)
Supplement: Supplementary Table S1 [file webappendixtables1word_mdx753.docx]

|  | **Appendix table S1: Major pathology criteria of the eligible patients (n=1226). The pathology criteria of the randomised versus the non-randomised patients were based on review pathology.** | | | | | | | | | | | |  |
| --- | --- | --- | --- | --- | --- | --- | --- | --- | --- | --- | --- | --- | --- |
|  |  |  |  |  |  |  |  |  |  |  |  |  |  |
|  |  |  |  |  |  |  |  |  |  |  |  |  |  |
|  |  |  |  |  |  | **Randomised eligible patients (N=329)** | |  | **Non-randomised eligible patients (N=897)** | |  |  |  |
|  | **Major pathologic criteria** | | | |  |  |  |  |  |  |  |  |  |
|  |  |  |  |  |  | **N** | **%** |  | **N** | **%** |  | **p-Value** |  |
|  | Age |  |  | < 60 |  | 141 | 43% |  | 198 | 25% |  |  |  |
|  |  |  |  | 60-69 |  | 136 | 41% |  | 347 | 44% |  |  |  |
|  |  |  |  | ≥ 70 |  | 52 | 16% |  | 249 | 31% |  |  |  |
|  |  |  |  | Missing |  | 0 |  |  | 103 |  |  | **<0.001** |  |
|  | FIGO stage (2009) | |  | IA |  | 43 | 13% |  | 167 | 19% |  |  |  |
|  |  |  |  | IB |  | 61 | 19% |  | 210 | 24% |  |  |  |
|  |  |  |  | II |  | 97 | 30% |  | 265 | 30% |  |  |  |
|  |  |  |  | IIIA |  | 48 | 15% |  | 92 | 11% |  |  |  |
|  |  |  |  | IIIB |  | 28 | 9% |  | 52 | 6% |  |  |  |
|  |  |  |  | IIIC |  | 51 | 16% |  | 90 | 10% |  |  |  |
|  |  |  |  | Missing |  | 1 |  |  | 21 |  |  | **0.002** |  |
|  | Histological type | |  | Endometrioid or mucinous |  | 207 | 63% |  | 556 | 63% |  |  |  |
|  |  |  |  | Serous or mixed serous |  | 70 | 21% |  | 189 | 21% |  |  |  |
|  |  |  |  | Clearcell or mixed clear cell |  | 43 | 13% |  | 99 | 11% |  |  |  |
|  |  |  |  | Other* |  | 9 | 3% |  | 45 | 5% |  |  |  |
|  |  |  |  | Missing |  | 0 |  |  | 8 |  |  | 0.295 |  |
|  | Histological grade | |  | EEC grade 1 |  | 63 | 19% |  | 173 | 20% |  |  |  |
|  |  |  |  | EEC grade 2 |  | 47 | 14% |  | 141 | 16% |  |  |  |
|  |  |  |  | EEC grade 3 |  | 95 | 29% |  | 233 | 26% |  |  |  |
|  |  |  |  | NEEC |  | 124 | 38% |  | 337 | 38% |  |  |  |
|  |  |  |  | Missing |  | 0 |  |  | 13 |  |  | 0.795 |  |
|  | Myometrial invasion | | | < 50% |  | 112 | 35% |  | 238 | 39% |  |  |  |
|  |  |  |  | ≥ 50 % |  | 206 | 65% |  | 373 | 61% |  |  |  |
|  |  |  |  | Missing |  | 11 |  |  | 286 |  |  | 0.414 |  |
|  | Growth through serosa | |  | Yes |  | 17 | 5% |  | 35 | 5% |  |  |  |
|  |  |  |  | No |  | 299 | 95% |  | 722 | 95% |  |  |  |
|  |  |  |  | Missing |  | 13 |  |  | 140 |  |  | 0.599 |  |
|  | Cervical glandular involvement | |  | Yes |  | 142 | 46% |  | 165 | 37% |  |  |  |
|  |  |  |  | No |  | 170 | 54% |  | 284 | 63% |  |  |  |
|  |  |  |  | Missing |  | 17 |  |  | 448 |  |  | 0.015 |  |
|  | Cervical stromal involvement | |  | Yes |  | 154 | 48% |  | 323 | 42% |  |  |  |
|  |  |  |  | No |  | 166 | 52% |  | 441 | 58% |  |  |  |
|  |  |  |  | Missing |  | 9 |  |  | 133 |  |  | 0.077 |  |
|  | LVSI |  |  | Yes |  | 186 | 58% |  | 299 | 57% |  |  |  |
|  |  |  |  | No |  | 135 | 42% |  | 228 | 43% |  |  |  |
|  |  |  |  | Missing |  | 8 |  |  | 370 |  |  | 0.695 |  |
|  | Involvement of the ovaries | |  | Yes |  | 58 | 18% |  | 55 | 7% |  |  |  |
|  |  |  |  | No |  | 265 | 82% |  | 723 | 93% |  |  |  |
|  |  |  |  | Missing |  | 6 |  |  | 119 |  |  | **<0.001** |  |
|  | Lymph node involvement | |  | Not applicable |  | 205 | 63% |  | 600 | 68% |  |  |  |
|  |  |  |  | No malignancy |  | 69 | 21% |  | 188 | 21% |  |  |  |
|  |  |  |  | Metastasis |  | 52 | 16% |  | 90 | 10% |  |  |  |
|  |  |  |  | Missing |  | 3 |  |  | 19 |  |  | 0.022 |  |
|  | Parametrial involvement | |  | Yes |  | 32 | 13% |  | 53 | 16% |  |  |  |
|  |  |  |  | No |  | 217 | 87% |  | 276 | 84% |  |  |  |
|  |  |  |  | Missing |  | 80 |  |  | 568 |  |  | 0.273 |  |
|  |  |  |  |  |  |  |  |  |  |  |  |  |  |
|  | Missing values were not taken into account to the percentages or the p-values. | | | | | | |  |  |  |  |  |  |
|  | Abbreviations: FIGO: International Federation of Gynecology and Obstetrics; LVSI: lymph-vascular space invasion; EEC: endometrioid endometrial cancer; NEEC: non-endometrioid endometrial cancer | | | | | | | | | | | |  |
|  |  |  |  |  |  |  |  |  |  |  |  |  |  |
|  | * other histology includes undifferentiated, other mixed combinations | | | | | | | | | | | |  |
|  |  |  |  |  |  |  |  |  |  |  |  |  |  |
