# Supplementary material for: Clinical consequences of upfront pathology review in the randomised PORTEC-3 trial for high-risk endometrial cancer
Source: Ann Oncol. 2017 Nov 27;29(2):424–30. doi: 10.1093/annonc/mdx753 (PMC5834053; doi:10.1093/annonc/mdx753)
Supplement: Supplementary Table S2 [file webappendixtables2word_mdx753.docx]

|  | **Appendix table S2: Inter-observer variability between original and review pathology report for the NL and UK cohorts separately.** | | | | | | |  |  |  |  |
| --- | --- | --- | --- | --- | --- | --- | --- | --- | --- | --- | --- |
|  |  |  |  |  |  |  |  |  |  |  |  |
|  | **NL cohort** |  |  |  |  |  |  |  |  |  |  |
|  | Pathology item | Total number available for analysis* | Missing items | Total discrepancies | Disagreement % ^#^ | Leading to ineligibility | Leading to ineligibility % ^$^ | Not leading to ineligibility | Not leading to ineligibility % ^¥^ | Kappa value |  |
|  | Histological type | 368 | 0 | 58 | 16% | 14 | 24% | 44 | 76% | 0.65 |  |
|  | Histological grade (EEC only) | 236 | 0 | 51 | 22% | 7 | 14% | 44 | 86% | 0.65 |  |
|  | Myometrial invasion | 366 | 2 | 33 | 9% | 3 | 9% | 30 | 91% | 0.80 |  |
|  | Cervical glandular involvement | 311 | 57 | 41 | 13% | 0 | 0% | 41 | 100% | 0.66 |  |
|  | Cervical stromal involvement | 357 | 11 | 33 | 9% | 12 | 36% | 21 | 64% | 0.80 |  |
|  | LVSI | 287 | 81 | 37 | 13% | 2 | 5% | 35 | 95% | 0.73 |  |
|  | Growth through serosa | 360 | 8 | 12 | 3% | 0 | 0% | 12 | 100% | 0.65 |  |
|  | Total | 2285 | 159 | 265 | 12% | 38 | 14% | 227 | 86% | NA |  |
|  |  |  |  |  |  |  |  |  |  |  |  |
|  |  |  |  |  |  |  |  |  |  |  |  |
|  | **UK cohort** |  |  |  |  |  |  |  |  |  |  |
|  | Pathology item | Total number available for analysis* | Missing items | Total discrepancies | Disagreement % ^#^ | Leading to ineligibility | Leading to ineligibility % ^$^ | Not leading to ineligibility | Not leading to ineligibility % ^¥^ | Kappa value |  |
|  | Histological type | 849 | 9 | 127 | 15% | 21 | 17% | 106 | 83% | 0.74 |  |
|  | Histological grade (EEC only) | 465 | 0 | 88 | 19% | 12 | 14% | 76 | 86% | 0.71 |  |
|  | Myometrial invasion | 557 | 301 | 55 | 10% | 4 | 7% | 51 | 93% | 0.79 |  |
|  | Cervical glandular involvement | 315 | 543 | 32 | 10% | 0 | 0% | 32 | 100% | 0.78 |  |
|  | Cervical stromal involvement | 706 | 152 | 36 | 5% | 15 | 42% | 21 | 58% | 0.90 |  |
|  | LVSI | 475 | 383 | 65 | 14% | 2 | 3% | 63 | 97% | 0.72 |  |
|  | Growth through serosa | 704 | 154 | 12 | 2% | 0 | 0% | 12 | 100% | 0.82 |  |
|  | Total (including serosal breach) | 4071 | 1542 | 415 | 10% | 54 | 13% | 361 | 87% | NA |  |
|  |  |  |  |  |  |  |  |  |  |  |  |
|  | * Total number of pathology items available for comparison between original and review pathology. # Total discrepancies / total number of pathology items available for analysis. $ number of pathology items leading to inelegibility / total discrepancies. ¥ number of pathology items not leading to ineligibility / total discrepancies. | | | | | | | | | |  |
|  |  |  |  |  |  |  |  |  |  |  |  |
|  | Abbreviations: LVSI; lymph vascular space invasion. EEC; endometrioid endometrial cancer | | | | | | | | | |  |
|  |  |  |  |  |  |  |  |  |  |  |  |
