# Supplementary material for: Clinical consequences of upfront pathology review in the randomised PORTEC-3 trial for high-risk endometrial cancer
Source: Ann Oncol. 2017 Nov 27;29(2):424–30. doi: 10.1093/annonc/mdx753 (PMC5834053; doi:10.1093/annonc/mdx753)
Supplement: Supplementary Data [file portec_3_study_group_and_participating_centres_mdx753.pdf]

PORTEC 3 Study group and participating centres

PORTEC-3 Independent data monitoring committee:
L.V.A.M. Beex, N. James, M.J.M. Olofsen-van Acht, W. Parulekar, W.L.J. van Putten, D. Rischin, J. Yarnold (chair)
PORTEC-3 trial statistician: H. Putter

List of participating countries and centres (listed in order of patients recruited):
United Kingdom: 184 patients (177 evaluable)
Study Coordinators: M. Powell (P.I.), London; H. Kitchener, Manchester; J. Ledermann, London
Group coordinating trial centre: Cancer Research UK and UCL Cancer Trials Centre, London
Trial pathologists: N. Singh, London; G. Wilson, Manchester
Participating centres and principal investigator(s) (number of patients):
London-Barts Health NHS Trust (M. Powell, 23); London-University College London Hospitals NHS Foundation Trust (M. McCormack, 15); Bebington, Wirral-The Clatterbridge Cancer Centre NHS Foundation Trust (K. Whitmarsh, K. Hyatt, 12); Wolverhampton-The Royal Wolverhampton NHS Trust (R. Allerton, 11); Cambridge-Cambridge University Hospitals NHS Foundation Trust (L.T. Tan, M. Iddawela, D. Gregory, S. Ayres, 11); Leicester-Leicester Royal Infirmary (P. Symonds, 10); Northwood-Mount Vernon Cancer Centre (P. Hoskin, 10); Middlesborough-The James Cook University Hospital (A. Rathmell, M. Adusumalli, 9); Nottingham-Nottingham City Hospital (S. Chan, A Anand, 9); Norwich-Norfolk and Norwich University Hospitals NHS Foundation Trust (R. Wade, 8); Guildford-Royal Surrey County Hospital (A. Stewart, 6); Newcastle Upon Tyne-Freeman Hospital (W. Taylor, 6); Brighton-Brighton & Sussex University Hospitals NHS Trust (K. Lankester, 5); Coventry-University Hospital Coventry (C. Irwin, M. Hocking, 5); Taunton-Musgrove Park Hospital (P. Jankowska, D. Milliken, C. Barlow, 5); Cheltenham-Cheltenham General Hospital (A. Cook, R. Counsell, 4); Exeter-Royal Devon & Exeter Hospital (P. Bliss, A. Hong, 4); Lincoln-Lincoln County Hospital (M. Panades, 4); Romford-Queens Hospital (M. Quigley, 4); Manchester-The Christie NHS Foundtion Trust (S. Davidson, 3); Northampton-Northampton General Hospital NHS Trust (C. Mak, 3); Preston-Royal Preston Hospital (A. Hindley, 3); Truro-Royal Cornwall Hospitals NHS Trust (A. Thomson, 3); Sheffield-Weston Park Hospital (S. Pledge, J. Martin, 2); Shrewsbury-Royal Shrewsbury Hospital (S. Awwad, A. Zachariah, 2); Carlisle-North Cumbria University Hospitals NHS Trust (S. Singhal, 1); Colchester-Essex County Hospital (A. Lamont, 1); London-Guy's and St. Thomas' NHS Foundation Trust (A. Winship, A. Montes, V. Mullassery, 1); London-Hammersmith Hospital - Imperial College Healthcare NHS Trust (A. Taylor, 1); Poole-Poole Hospital NHS Foundation Trust (V. Laurence, M. Flubacher, 1); Reading-Royal Berkshire Hospital (H. O'Donnell, 1); Stoke-on-Trent-Royal Stoke University Hospital (R. Bhana, S. Lupton, 1)

The Netherlands: 145 patients (138 evaluable)
Study Coordinators: C.L. Creutzberg (C.I.), Leiden; R. Kruitwagen, Maastricht; H. Nijman, Groningen; N. Ottevanger, Nijmegen
Group coordinating trial centre: Netherlands Comprehensive Cancer Organisation (IKNL), Leiden
Trial pathologists: H. Hollema, Groningen; V.T. Smit, Leiden
Participating centres and principal investigator(s) (number of patients):
University Medical Center Utrecht (I.M. Jurgenliemk-Schulz, 20); Maastro Clinic, Maastricht (L.C.H.W. Lutgens, 17); University Medical Center Groningen (E. Pras, 15); Leiden University Medical Center (C.L. Creutzberg, R. Nout, 15); University Medical Center Radboud, Nijmegen (J.W.H. Leer, A. Snyers, 11); Academic Medical Center, Amsterdam (A.L.J. Uitterhoeve, L. Stalpers, 9); Medical Spectre Twente, Enschede (J.J. Jobsen, 9); Radiotherapy institute Friesland, Leeuwarden (A. Slot, 9); Erasmus Medical Center Rotterdam (J.W.M. Mens, 8); Medical Center Haaglanden/ Radiotherapy Centre West (T.C. Stam, P.C.M. Koper, 7); Netherlands Cancer Institute, Amsterdam (B. van Triest, 6); Radiotherapy Group, Arnhem (E.M. van der Steen-Banasik, 6); Radiotherapy Institute Verbeeten, Tilburg (K.A.J. de Winter, 6); Radiotherapy Group, Deventer (S. van de Pol, 3); Catharina Hospital, Eindhoven (H.A. van den Berg, 3); VU Medical Centre, Amsterdam (O.W.M. Meijer, 1)

Australia & New Zealand: 122 patients (118 evaluable)
ANZGOG Study Coordinators: L. Mileshkin (P.I.) Melbourne; M. Quinn, Melbourne; P. Khaw Melbourne; I.Kolodziej, Sydney

Group coordinating trial centre: NHMRC Clinical Trials Centre, Sydney
Trial pathologist: J.Pyman, Melbourne
Participating centres and principal investigator(s) (number of patients):
Peter MacCallum Cancer Centre, Melbourne, Victoria, Australia (L. Mileshkin, P.Khaw; 31); Monash Cancer Centre (Monash Medical Centre), East Bentleigh, Victoria, Australia (P. Khaw/G. Goss, 20); Westmead Hospital, Wentworthville, NSW, Australia (G. Wain, 15); Auckland City Hospital , Auckland, New Zealand (S. Brooks, 13); Wellington Blood & Cancer Centre, Wellington, New Zealand (C. Johnson, 11); Calvary Mater Newcastle, Newcastle, Australia (A. Capp, 8); Christchurch Hospital, Canterbury, New Zealand (M. Vaughan, 4); Royal Hobart Hospital, Hobart, Tasmania, Australia (P. Blomfield, 3); Palmerston North Hospital, Palmerston North, New Zealand (C. Hardie, 3); Royal North Shore Hospital, St Leonards, NSW, Australia (M. Stevens, 3); Waikato Hospital, Hamilton, New Zealand (M. Kuper, 2); Royal Brisbane &Women's Hospital, Brisbane, QLD, Australia(R. Cheuk, 2); Liverpool Hospital, Liverpool, NSW, Australia (S. Vinod, 2); Mater Hospital Brisbane , South Brisbane, QLD, Australia(C. Shannon/J. Ramsay, 2); Wollongong Hospital, Wollongong, NSW, Australia (A. Glasgow, 2); Townsville Hospital, Townsville, QLD, Australia (S. Hewitt, 1)

Italy: 103 patients (98 evaluable)
MaNGO Study Coordinators: R. Fossati (P.I.) Milano; D. Katsaros, Torino; A. Colombo, Lecco
Group coordinating trial centre: Istituto di Ricerche Farmacologiche Mario Negri, Milano
Trial pathologists: S. Carinelli, Milano; C. Di Tonno, Milano
Participating centres and principal investigator(s) (number of patients):
Torino - S. Anna Hospital (S. Gribaudo, M. Mitidieri, 33); Lecco - Ospedale A. Manzoni (R. D'Amico, 24); Monza - S. Gerardo Hospital (S. Meregalli, A. A. Lissoni, 16); Torino - Ospedale Umberto I (A. Ferrero, 7); Padova – Istituto Oncologico Veneto / Mirano, Venezia – Azienda ULSS 13 (L. Corti, G. Artioli, 6); Varese - H. Del Ponte University of Insubria (C. Apolloni, 4); Ravenna - Ospedale S. Maria delle Croci (D. Turci, 4); Brescia – Spedali Civili (G. Tognon, 2); Como – ASST Lariana Ospedale S. Anna (E. Bianchi, 2); Meldola - Istituto Scientifico Romagnolo per lo Studio e la Cura dei Tumori (E. Bianchi, 2); Genova – IRCCS San Martino IST (M. Bruzzone, 1); Milano - ASST Grande Ospedale Metropolitano Niguarda (S. Siena, 1); Palermo – AOR Villa Sofia-Cervello (N. Varsellona, 1)

Canada: 65 patients (65 evaluable)
CCTG Study Coordinators: A. Fyles Toronto, Ontario; P. Bessette, Sherbrooke, Quebec
Group coordinating trial centre: Canadian Cancer Trials Group, Kingston, Ontario
Trial pathologist: M. McLachlin, London, Ontario
Participating centres and principal investigator(s) (number of patients):
Sherbrooke-Centre Hosp. Universitaire de Sherbrooke (P. Bessette, 18); Montreal-Hopital Notre-Dame de Montreal (D. Provencher, 11); Calgary-Tom Baker Cancer Centre (P. Ghatage, 9); Halifax-Queen Elizabeth II Health Sciences Centr (P. Rittenberg, 8); Montreal-McGill Oncology Montreal (L. Souhami, 7); Toronto-Sunnybrook Health Sciences Centre (G. Thomas, 7); Quebec-Hotel-Dieu de Quebec (M. Plante, 2); London-London Health Sciences Centre (A. Hammond, 1); St John's-Dr. H. Bliss Murphy Cancer Centre (P. Power, 1); Toronto-Princess Margaret Hospital (A. Fyles, 1)

France: 67 patients (64 evaluable)
FEDEGYN Study Coordinator: Chr. Haie-Meder (P.I.) Paris
Group coordinating trial centre: UNICANCER, Paris
Trial pathologist: P. Duvillard, Paris
Participating centres and principal investigator(s) (number of patients):
Besancon-Hopital Jean Minjoz (M-H Baron, 10); Rouen-Centre Henri Becquerel (Hanzen, 9); Saint Herblain-Centre Rene Gauducheau (D. Berton-Rigaud, 8); Limoges-CHU Limoges (Pr. N. Tubiana-Mathieu, 6); Bordeaux-Institut Bergonie (L. Thomas, 5); Reims-Institut Jean Godinot (A. Savoye, S. Maillard, 5); Dijon-Centre Georges Francois Leclerc (K. Peignaux, 4); Paris/ Villejuif-Institut Gustave Roussy (C. Haie Meder, 4); Clermont-Ferrand-Centre Jean Perrin (C. Benoit, 3); Montpellier-Centre Val d'Aurelle (C. Kerr, 3); Toulouse Cedex-Institut Claudius Regaud (L. Gladieff, 3); Caen-Centre Francois Baclesse (D. Lerouge, 2); Nice Cedex-Centre Antoine Lacassagne (P. Follana, 2); Marseille-Institut Paoli Calmettes (M. Cappiello, 1); Strasbourg-Centre Paul Strauss (T. Petit, 1); Tours Cedex-CHU de Tours - Hopital Bretonneau (I. Barillot, 1)
